# (4-Chloro-2-fluoro­phen­yl)[1-(2,6-difluoro­phen­yl)but-3-en­yl]amine

**DOI:** 10.1107/S1600536809012896

**Published:** 2009-04-10

**Authors:** Hoong-Kun Fun, Sankappa Rai, Prakash Shetty, Arun M. Isloor, Suchada Chantrapromma

**Affiliations:** a X-ray Crystallography Unit, School of Physics, Universiti Sains Malaysia, 11800 USM, Penang, Malaysia; b Syngene International Ltd., Biocon Park, Plot No. 2&3, Bommasandra 4th Phase, Jigani Link Road, Bangalore 560 100, India; c Department of Printing, Manipal Institute of Technology, Manipal 576 104, India; d Department of Chemistry, National Institute of Technology–Karnataka, Surathkal, Mangalore 575 025, India; e Crystal Materials Research Unit, Department of Chemistry, Faculty of Science, Prince of Songkla University, Hat-Yai, Songkhla 90112, Thailand

## Abstract

In the mol­ecule of the title homoallylic amine, C_16_H_13_ClF_3_N, the dihedral angle between the two benzene rings is 84.63 (4)°. Weak intra­molecular N—H⋯F hydrogen bonds generate *S*(6) and *S*(5) ring motifs. In the crystal structure, weak inter­molecuar N—H⋯F hydrogen bonds link mol­ecules into centrosymmetric dimers which are arranged in mol­ecular sheets parallel to the *ac* plane.

## Related literature

For standard bond lengths, see Allen *et al.* (1987 ▶). For hydrogen-bond motifs, see Bernstein *et al.* (1995 ▶). For background to the bioactivity and applications of homoallylic amines, see: Edwards *et al.* (1998 ▶); Robert (1998 ▶); Sabine & Horst (1991 ▶); Xie *et al.* (1989 ▶). For the stability of the temperature controller used in the data collection, see: Cosier & Glazer (1986 ▶).
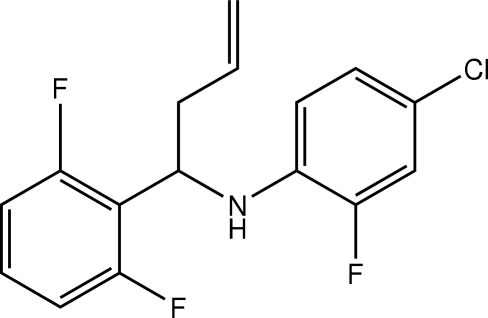


## Experimental

### 

#### Crystal data

C_16_H_13_ClF_3_N
*M* *_r_* = 311.72
Monoclinic, 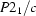

*a* = 10.8980 (1) Å
*b* = 14.0073 (2) Å
*c* = 10.1651 (1) Å
β = 113.018 (1)°
*V* = 1428.17 (3) Å^3^
*Z* = 4
Mo *K*α radiation
μ = 0.29 mm^−1^
*T* = 100 K
0.50 × 0.39 × 0.27 mm

#### Data collection

Bruker APEXII CCD area-detector diffractometer
Absorption correction: multi-scan (*SADABS*; Bruker, 2005 ▶) *T*_min_ = 0.868, *T*_max_ = 0.926
32850 measured reflections
7434 independent reflections
6099 reflections with *I* > 2σ(*I*)
*R*_int_ = 0.025

#### Refinement

*R*[*F* ^2^ > 2σ(*F* ^2^)] = 0.041
*wR*(*F* ^2^) = 0.119
*S* = 1.04
7434 reflections
202 parameters
H atoms treated by a mixture of independent and constrained refinement
Δρ_max_ = 0.63 e Å^−3^
Δρ_min_ = −0.86 e Å^−3^

### 

Data collection: *APEX2* (Bruker, 2005 ▶); cell refinement: *SAINT* (Bruker, 2005 ▶); data reduction: *SAINT*; program(s) used to solve structure: *SHELXTL* (Sheldrick, 2008 ▶); program(s) used to refine structure: *SHELXTL*; molecular graphics: *SHELXTL*; software used to prepare material for publication: *SHELXTL* and *PLATON* (Spek, 2009 ▶).

## Supplementary Material

Crystal structure: contains datablocks global, I. DOI: 10.1107/S1600536809012896/lh2803sup1.cif

Structure factors: contains datablocks I. DOI: 10.1107/S1600536809012896/lh2803Isup2.hkl

Additional supplementary materials:  crystallographic information; 3D view; checkCIF report

## Figures and Tables

**Table 1:** Hydrogen-bond geometry (Å, °)

*D*—H⋯*A*	*D*—H	H⋯*A*	*D*⋯*A*	*D*—H⋯*A*
N1—H1*N*1⋯F1	0.886 (17)	2.510 (14)	2.8354 (9)	102.4 (11)
N1—H1*N*1⋯F3	0.886 (17)	2.306 (17)	2.6839 (9)	105.7 (14)
N1—H1*N*1⋯F1^i^	0.886 (17)	2.194 (17)	3.0639 (9)	167.1 (16)
C7—H7*A*⋯F2	0.98	2.38	2.8330 (10)	107

Symmetry code: (i) 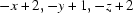.

**Fig. 1.**
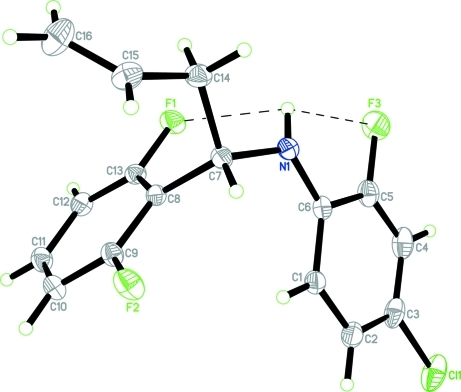
The molecluar structure of (I), showing 50% probability displacement ellipsoids and the atom-numbering scheme. Hydrogen bonds are drawn as dash lines.

**Fig. 2.**
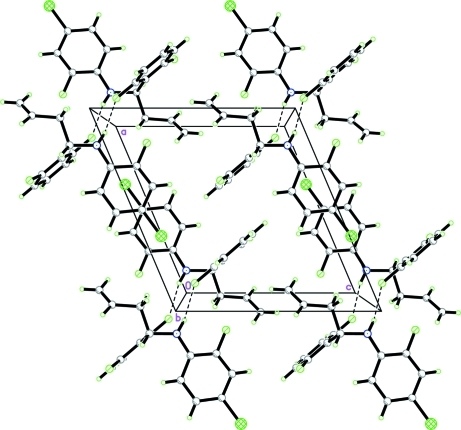
Part of the crystal structure of (I), viewed along the b axis, showing the arrangement of the hydrogen-bonded dimers into molecular sheets. Hydrogen bonds are shown as dashed lines.

## supplementary crystallographic information

### Comment

Homoallylic amines are valuable intermediates in organic synthesis and as starting materials in the preparation of biologically active substances, resolving agents and chiral auxillaries for asymmetric synthesis (Sabine & Horst, 1991) and synthesis of β-amino acids (Xie *et al.*, 1989), β-lactams (Edwards *et al.*, 1998) and HIV-proteaseinhibitors (Robert, 1998). Prompted by these observations, we have synthesized the title compound and its crystal structure is presented herein.

In the molecular structure of the title homoallylic amine (I) (Fig. 1), angle between the mean planes of the benzene rings is 84.63 (4)°. The orientation of the but-3-enyl substituent group [C7/C14–C16] with respect to the 2,6-difluoro-phenyl ring is reflected in the torsion angle C8–C7–C14–C15 = 59.43 (9)° which indicates a (+)-*syn*-clinal conformation. The torsion angle C7–C14–C15–C16 = -122.17 (11)°. The bond distances in (I) have normal values (Allen *et al.*, 1987).

In the structure, intramolecular N1—H1N1···F1 and N1—H1N1···F3 hydrogen bonds generate S(6) and S(5) ring motifs, respectively (Bernstein *et al.*, 1995) (Table 1). In the crystal structure, weak N—H···F hydrogen bonds (Table 1, Fig. 2) link molecules into centrosymmetric dimers and these dimers are arranged into molecular sheets parallel to the *ac* plane.

### Experimental

To a mixture of 2,6-difluorobenzaldehyde (0.5 g, 3.5 mmol), 4-chloro-2-fluoro aniline (0.51 g, 3.5 mmol) and allyltributyltin (1.1 g, 3.5 mmol) in acetonitrile (5 ml), trifluoro acetic acid (0.04 g, 0.35 mmol) was added. The reaction mixture was stirred at 299 K under nitrogen atmosphere for 2 h. Completion of the reaction was monitored by TLC. The reaction mixture was then extracted with diethyl ether (3 *x* 20 ml) and the combined organic layer were concentrated in vacuum and purified by flash chromatography to afford the pure homoallylic amine. The yield was found to be 0.98 g (90% yield). Colorless block-shaped single crystals of the title compound was recrystalized in acetone by slow evaporation of the solvent, *M*.p 399–400 K.

### Refinement

Amine and =CH_2_ H atoms were located from the difference map and refined isotropically. The remaining H atoms were placed in calculated positions with d(C—H) = 0.93 Å, *U*_iso_ = 1.2*U*_eq_(C) for aromatic and 0.98 Å, *U*_iso_ = 1.2*U*_eq_(C) for CH. The highest residual electron density peak is located at 0.63 Å from Cl1 and the deepest hole is located at 0.62 Å from Cl1.

### Crystal data

**Table:**

C_16_H_13_ClF_3_N	*F*(000) = 640
*M**_r_* = 311.72	*D*_x_ = 1.450 Mg m^−^^3^
Monoclinic, *P*2_1_/*c*	Melting point = 399–400 K
Hall symbol: -P 2ybc	Mo *K*α radiation, λ = 0.71073 Å
*a* = 10.8980 (1) Å	Cell parameters from 7434 reflections
*b* = 14.0073 (2) Å	θ = 2.0–37.5°
*c* = 10.1651 (1) Å	µ = 0.29 mm^−^^1^
β = 113.018 (1)°	*T* = 100 K
*V* = 1428.17 (3) Å^3^	Block, colorless
*Z* = 4	0.50 × 0.39 × 0.27 mm

### Data collection

**Table:**

Bruker APEXII CCD area-detector diffractometer	7434 independent reflections
Radiation source: sealed tube	6099 reflections with *I* > 2σ(*I*)
graphite	*R*_int_ = 0.025
φ and ω scans	θ_max_ = 37.5°, θ_min_ = 2.0°
Absorption correction: multi-scan (*SADABS*; Bruker, 2005)	*h* = −15→18
*T*_min_ = 0.868, *T*_max_ = 0.926	*k* = −22→23
32850 measured reflections	*l* = −17→14

### Refinement

**Table:**

Refinement on *F*^2^	Primary atom site location: structure-invariant direct methods
Least-squares matrix: full	Secondary atom site location: difference Fourier map
*R*[*F*^2^ > 2σ(*F*^2^)] = 0.041	Hydrogen site location: inferred from neighbouring sites
*wR*(*F*^2^) = 0.119	H atoms treated by a mixture of independent and constrained refinement
*S* = 1.04	*w* = 1/[σ^2^(*F*_o_^2^) + (0.059*P*)^2^ + 0.3248*P*] where *P* = (*F*_o_^2^ + 2*F*_c_^2^)/3
7434 reflections	(Δ/σ)_max_ = 0.001
202 parameters	Δρ_max_ = 0.63 e Å^−^^3^
0 restraints	Δρ_min_ = −0.85 e Å^−^^3^

### Special details

**Table:**

Experimental. The crystal was placed in the cold stream of an Oxford Cryosystems Cobra open-flow nitrogen cryostat (Cosier & Glazer, 1986) operating at 100.0 (1) K.
Geometry. All e.s.d.'s (except the e.s.d. in the dihedral angle between two l.s. planes) are estimated using the full covariance matrix. The cell e.s.d.'s are taken into account individually in the estimation of e.s.d.'s in distances, angles and torsion angles; correlations between e.s.d.'s in cell parameters are only used when they are defined by crystal symmetry. An approximate (isotropic) treatment of cell e.s.d.'s is used for estimating e.s.d.'s involving l.s. planes.
Refinement. Refinement of *F*^2^ against ALL reflections. The weighted *R*-factor *wR* and goodness of fit *S* are based on *F*^2^, conventional *R*-factors *R* are based on *F*, with *F* set to zero for negative *F*^2^. The threshold expression of *F*^2^ > σ(*F*^2^) is used only for calculating *R*-factors(gt) *etc*. and is not relevant to the choice of reflections for refinement. *R*-factors based on *F*^2^ are statistically about twice as large as those based on *F*, and *R*- factors based on ALL data will be even larger.

### Fractional atomic coordinates and isotropic or equivalent isotropic displacement parameters (Å^2^)

**Table:**

	*x*	*y*	*z*	*U*_iso_*/*U*_eq_	
Cl1	0.35924 (3)	0.625873 (17)	1.05066 (4)	0.03621 (8)	
F1	0.89730 (5)	0.44888 (4)	0.88286 (5)	0.02227 (11)	
F2	0.65542 (6)	0.64050 (4)	0.48567 (6)	0.02478 (12)	
F3	0.85689 (7)	0.60562 (5)	1.18096 (6)	0.03051 (13)	
N1	0.84154 (7)	0.64304 (5)	0.91602 (7)	0.01799 (11)	
C1	0.59745 (8)	0.65594 (6)	0.83617 (9)	0.01870 (13)	
H1A	0.5873	0.6695	0.7429	0.022*	
C2	0.48485 (9)	0.65290 (6)	0.86995 (10)	0.02241 (15)	
H2A	0.4007	0.6643	0.7996	0.027*	
C3	0.49921 (10)	0.63283 (6)	1.00871 (11)	0.02434 (16)	
C4	0.62453 (11)	0.61733 (6)	1.11567 (10)	0.02539 (17)	
H4A	0.6347	0.6051	1.2093	0.030*	
C5	0.73310 (9)	0.62070 (6)	1.07837 (9)	0.02114 (14)	
C6	0.72486 (8)	0.63897 (5)	0.93977 (8)	0.01658 (12)	
C7	0.84187 (7)	0.64023 (5)	0.77318 (8)	0.01602 (12)	
H7A	0.7900	0.6949	0.7199	0.019*	
C8	0.78026 (7)	0.55017 (5)	0.68918 (7)	0.01418 (11)	
C9	0.69022 (8)	0.55353 (5)	0.54747 (8)	0.01668 (12)	
C10	0.63233 (8)	0.47417 (6)	0.46594 (8)	0.01959 (13)	
H10A	0.5721	0.4806	0.3717	0.024*	
C11	0.66630 (9)	0.38468 (6)	0.52828 (9)	0.02012 (14)	
H11A	0.6285	0.3303	0.4754	0.024*	
C12	0.75652 (8)	0.37582 (5)	0.66927 (9)	0.01879 (13)	
H12A	0.7801	0.3161	0.7117	0.023*	
C13	0.81007 (7)	0.45824 (5)	0.74445 (8)	0.01578 (12)	
C14	0.98669 (8)	0.65337 (6)	0.78678 (9)	0.02065 (14)	
H14A	1.0232	0.7107	0.8416	0.025*	
H14B	1.0396	0.5996	0.8389	0.025*	
C15	0.99758 (9)	0.66087 (6)	0.64501 (10)	0.02264 (15)	
H15A	0.9501	0.7093	0.5836	0.027*	
C16	1.06994 (12)	0.60355 (8)	0.60087 (13)	0.0329 (2)	
H1N1	0.9099 (16)	0.6141 (10)	0.9828 (17)	0.033 (4)*	
H16B	1.1209 (16)	0.5526 (12)	0.6600 (17)	0.041 (4)*	
H16A	1.0756 (18)	0.6098 (12)	0.513 (2)	0.049 (5)*	

### Atomic displacement parameters (Å^2^)

**Table:**

	*U*^11^	*U*^22^	*U*^33^	*U*^12^	*U*^13^	*U*^23^
Cl1	0.04381 (15)	0.02600 (11)	0.05793 (18)	−0.00690 (9)	0.04060 (14)	−0.00647 (10)
F1	0.0223 (2)	0.0231 (2)	0.0163 (2)	0.00354 (17)	0.00191 (17)	0.00387 (17)
F2	0.0323 (3)	0.0181 (2)	0.0175 (2)	0.00117 (18)	0.0027 (2)	0.00436 (17)
F3	0.0343 (3)	0.0376 (3)	0.0167 (2)	0.0065 (2)	0.0068 (2)	0.0050 (2)
N1	0.0171 (3)	0.0222 (3)	0.0138 (2)	0.0005 (2)	0.0051 (2)	−0.0018 (2)
C1	0.0189 (3)	0.0205 (3)	0.0177 (3)	−0.0013 (2)	0.0083 (2)	−0.0033 (2)
C2	0.0216 (4)	0.0214 (3)	0.0271 (4)	−0.0026 (3)	0.0127 (3)	−0.0062 (3)
C3	0.0312 (4)	0.0177 (3)	0.0338 (4)	−0.0045 (3)	0.0233 (4)	−0.0050 (3)
C4	0.0398 (5)	0.0197 (3)	0.0243 (4)	−0.0011 (3)	0.0208 (4)	0.0002 (3)
C5	0.0286 (4)	0.0187 (3)	0.0172 (3)	0.0009 (3)	0.0101 (3)	0.0003 (2)
C6	0.0199 (3)	0.0149 (3)	0.0159 (3)	−0.0008 (2)	0.0080 (2)	−0.0023 (2)
C7	0.0162 (3)	0.0165 (3)	0.0151 (3)	−0.0012 (2)	0.0059 (2)	−0.0011 (2)
C8	0.0144 (3)	0.0153 (3)	0.0132 (3)	0.0001 (2)	0.0057 (2)	0.0001 (2)
C9	0.0193 (3)	0.0159 (3)	0.0141 (3)	0.0004 (2)	0.0057 (2)	0.0012 (2)
C10	0.0214 (3)	0.0205 (3)	0.0148 (3)	−0.0019 (2)	0.0049 (2)	−0.0024 (2)
C11	0.0224 (3)	0.0178 (3)	0.0206 (3)	−0.0028 (2)	0.0089 (3)	−0.0038 (2)
C12	0.0209 (3)	0.0155 (3)	0.0213 (3)	0.0006 (2)	0.0096 (3)	0.0007 (2)
C13	0.0148 (3)	0.0177 (3)	0.0146 (3)	0.0016 (2)	0.0054 (2)	0.0019 (2)
C14	0.0166 (3)	0.0239 (3)	0.0214 (3)	−0.0038 (2)	0.0075 (3)	−0.0009 (3)
C15	0.0211 (3)	0.0241 (3)	0.0254 (4)	−0.0005 (3)	0.0120 (3)	0.0054 (3)
C16	0.0365 (5)	0.0360 (5)	0.0357 (5)	0.0056 (4)	0.0245 (4)	0.0063 (4)

### Geometric parameters (Å, °)

**Table:**

Cl1—C3	1.7398 (9)	C7—H7A	0.9800
F1—C13	1.3618 (9)	C8—C9	1.3910 (10)
F2—C9	1.3550 (9)	C8—C13	1.3915 (10)
F3—C5	1.3608 (11)	C9—C10	1.3826 (11)
N1—C6	1.3851 (11)	C10—C11	1.3879 (11)
N1—C7	1.4539 (10)	C10—H10A	0.9300
N1—H1N1	0.886 (16)	C11—C12	1.3907 (12)
C1—C6	1.3964 (11)	C11—H11A	0.9300
C1—C2	1.3983 (12)	C12—C13	1.3816 (11)
C1—H1A	0.9300	C12—H12A	0.9300
C2—C3	1.3857 (14)	C14—C15	1.4953 (12)
C2—H2A	0.9300	C14—H14A	0.9700
C3—C4	1.3904 (15)	C14—H14B	0.9700
C4—C5	1.3773 (13)	C15—C16	1.3208 (14)
C4—H4A	0.9300	C15—H15A	0.9300
C5—C6	1.3999 (11)	C16—H16B	0.958 (17)
C7—C8	1.5242 (10)	C16—H16A	0.924 (19)
C7—C14	1.5406 (11)		
			
C6—N1—C7	122.26 (7)	C13—C8—C7	123.88 (6)
C6—N1—H1N1	113.7 (10)	F2—C9—C10	117.74 (7)
C7—N1—H1N1	115.1 (10)	F2—C9—C8	117.83 (6)
C6—C1—C2	121.26 (8)	C10—C9—C8	124.42 (7)
C6—C1—H1A	119.4	C9—C10—C11	118.34 (7)
C2—C1—H1A	119.4	C9—C10—H10A	120.8
C3—C2—C1	119.70 (9)	C11—C10—H10A	120.8
C3—C2—H2A	120.2	C10—C11—C12	120.40 (7)
C1—C2—H2A	120.2	C10—C11—H11A	119.8
C2—C3—C4	120.87 (8)	C12—C11—H11A	119.8
C2—C3—Cl1	120.00 (8)	C13—C12—C11	118.10 (7)
C4—C3—Cl1	119.13 (7)	C13—C12—H12A	120.9
C5—C4—C3	117.80 (8)	C11—C12—H12A	120.9
C5—C4—H4A	121.1	F1—C13—C12	117.66 (7)
C3—C4—H4A	121.1	F1—C13—C8	117.69 (7)
F3—C5—C4	118.99 (8)	C12—C13—C8	124.64 (7)
F3—C5—C6	116.99 (8)	C15—C14—C7	112.74 (7)
C4—C5—C6	124.01 (8)	C15—C14—H14A	109.0
N1—C6—C1	124.85 (7)	C7—C14—H14A	109.0
N1—C6—C5	118.75 (7)	C15—C14—H14B	109.0
C1—C6—C5	116.34 (8)	C7—C14—H14B	109.0
N1—C7—C8	114.16 (6)	H14A—C14—H14B	107.8
N1—C7—C14	108.04 (6)	C16—C15—C14	124.58 (9)
C8—C7—C14	111.13 (6)	C16—C15—H15A	117.7
N1—C7—H7A	107.8	C14—C15—H15A	117.7
C8—C7—H7A	107.8	C15—C16—H16B	121.1 (10)
C14—C7—H7A	107.8	C15—C16—H16A	123.1 (11)
C9—C8—C13	114.10 (6)	H16B—C16—H16A	115.8 (14)
C9—C8—C7	122.01 (6)		
			
C6—C1—C2—C3	0.07 (12)	N1—C7—C8—C13	−47.16 (10)
C1—C2—C3—C4	1.19 (12)	C14—C7—C8—C13	75.34 (9)
C1—C2—C3—Cl1	−178.34 (6)	C13—C8—C9—F2	179.74 (7)
C2—C3—C4—C5	−1.30 (12)	C7—C8—C9—F2	−1.57 (11)
Cl1—C3—C4—C5	178.24 (6)	C13—C8—C9—C10	0.69 (11)
C3—C4—C5—F3	−179.88 (7)	C7—C8—C9—C10	179.38 (7)
C3—C4—C5—C6	0.17 (13)	F2—C9—C10—C11	−179.51 (8)
C7—N1—C6—C1	−16.99 (11)	C8—C9—C10—C11	−0.46 (13)
C7—N1—C6—C5	165.95 (7)	C9—C10—C11—C12	−0.06 (13)
C2—C1—C6—N1	−178.25 (7)	C10—C11—C12—C13	0.28 (12)
C2—C1—C6—C5	−1.12 (11)	C11—C12—C13—F1	179.36 (7)
F3—C5—C6—N1	−1.62 (11)	C11—C12—C13—C8	−0.01 (12)
C4—C5—C6—N1	178.32 (8)	C9—C8—C13—F1	−179.82 (6)
F3—C5—C6—C1	−178.93 (7)	C7—C8—C13—F1	1.51 (11)
C4—C5—C6—C1	1.02 (12)	C9—C8—C13—C12	−0.44 (11)
C6—N1—C7—C8	−59.79 (9)	C7—C8—C13—C12	−179.11 (7)
C6—N1—C7—C14	176.04 (7)	N1—C7—C14—C15	−174.60 (7)
N1—C7—C8—C9	134.28 (7)	C8—C7—C14—C15	59.43 (9)
C14—C7—C8—C9	−103.22 (8)	C7—C14—C15—C16	−122.17 (11)

### Hydrogen-bond geometry (Å, °)

**Table:**

*D*—H···*A*	*D*—H	H···*A*	*D*···*A*	*D*—H···*A*
N1—H1N1···F1	0.886 (17)	2.510 (14)	2.8354 (9)	102.4 (11)
N1—H1N1···F3	0.886 (17)	2.306 (17)	2.6839 (9)	105.7 (14)
N1—H1N1···F1^i^	0.886 (17)	2.194 (17)	3.0639 (9)	167.1 (16)
C7—H7A···F2	0.98	2.38	2.8330 (10)	107

Symmetry codes:  (i) −*x*+2, −*y*+1, −*z*+2.
